# Subjective Dry Eye Symptoms in Pregnant Women–A SPEED Survey

**DOI:** 10.1155/2023/3421269

**Published:** 2023-01-04

**Authors:** Divya Anantharaman, Aiswaryah Radhakrishnan, Vidhyalakshmi Anantharaman

**Affiliations:** Department of Ophthalmology - Optometry, SRM Medical College Hospital and Research Centre, Faculty of Medicine and Health Sciences, SRM Institute of Science and Technology, SRM Nagar, Kattankulathur-603203, Kanchipuram, Chennai, India

## Abstract

**Aim:**

Multisystemic physiological changes in pregnancy can result in tear film and refractive changes in the eye. We report dry eye prevalence in pregnant women using Standard Patient Evaluation of Eye Dryness (SPEED) questionnaire.

**Methods:**

The SPEED questionnaire was self-administered cross-sectionally to 428 pregnant women (mean age: 26.8 ± 4.4 years) with clinically confirmed pregnancy from two obstetric clinics in Chennai, India. Subjects with predisposing risk factors for dry eye were excluded from the study. Subjects were categorized as normal, moderate, and severe dry eye based on the SPEED score.

**Results:**

Among the women, 48.5% of the subjects had symptoms like dryness, grittiness or scratchiness, soreness or irritation, burning or watering, or eye fatigue. About 2.3% had moderate dry eye according to SPEED questionnaire criteria. Eye fatigue was the most reported symptom and was present in 76.4% of women. The symptom frequency score and severity score had a strong and significant correlation (*r* = 0.95, *P* < .001). No significant correlation was noted among SPEED score vs age (*r* = −0.02, *P* > .05). No significant correlation was found between symptoms of dry eye and gravidity (*ρ* = −0.006, *P* > .05) and trimester (*ρ* = 0.38, *P* > .05). Binary logistic regression showed that only occupational status and systemic condition was significantly associated with dry eye symptoms.

**Conclusion:**

About half the pregnant women at the visit reported having one or more dry eye-related symptoms. As per the composite SPEED questionnaire score, dry eye was not prevalent among pregnant women irrespective of their age, gravidity, and the trimester, but we found a majority of pregnant women reported to have experienced dry eye-related symptoms, though tolerable. Awareness about dry eye during pregnancy will improve eye care seeking behaviour in pregnant women.

## 1. Introduction

Dry eye is a multifactorial ocular surface disorder characterized by a loss of homeostasis of the tear film, resulting in tear film instability, and ocular surface inflammation/damage [1]. While the worldwide prevalence of dry eye ranges between 5% and 50%, in India it is reported to be 15.4%-45.4% [2–6]. Several risk factors for dry eye, including older age, females, Asian ethnicity, contact lens wear, and hormonal imbalances have been reported. However, pregnancy as a specific risk factor for dry eye has not been well studied [1, 2, 7].

Studies report that during pregnancy, lacrimal gland growth factor expression can be altered and lacrimal acinar cells could be damaged, increasing the risk of dry eye [8, 9]. On the other hand, positive objective findings of dry eye among pregnant women with the absence of subjective findings are also reported [10–14]. Women undergo significant emotional and physiological changes during pregnancy [15, 16]. Identifying and alleviating the symptoms related to dry eye can potentially improve the quality of life in pregnant women [2, 13, 17–21]. Only a few studies are available on dry eye prevalence in India, and none that address the prevalence of dry eye during pregnancy [6].

Earlier studies have identified the Standard Patient Evaluation of Eye Dryness (SPEED) questionnaire as a standardized valid instrument for identifying dry eye symptoms, its frequency and severity [22–24]. Ngo et al. has reported that the SPEED score correlates significantly with ocular surface staining and clinical measures of Meibomian gland function [22]. Asiedu et al. in a nonclinical sample cross sectional study, it was noted that SPEED had better performance in separating asymptomatic and symptomatic participants in comparison with the Ocular Surface Disease Index (OSDI) questionnaire [25]. Thus, in this study, we investigated the prevalence of subjective dry eye symptoms among pregnant women by self-administration of the SPEED questionnaire.

## 2. Methods

In this cross-sectional study, 428 women (Mean age: 26.8 ± 4.4 years, between 18-40 years of age) with clinically confirmed pregnancy (positive blood/urine analysis or pelvic ultrasonography) were included from the obstetric outpatient departments of two hospitals in Chennai, India. Participants with predisposing dry eye risk factors such as Sjogren's syndrome, history of any ocular surgery, lid abnormality, facial nerve palsy, contact lens wear, and more than 7 hours of computer usage were excluded from the study. The study followed the protocols recommended by the tenets of the Declaration of Helsinki and the methods were approved by the Institutional Ethical Committee. All participants provided written informed consent.

Paper-based SPEED questionnaire administration was done for the participants. The participants' indicated the presence or absence, the frequency, and the severity of four dry eye-related symptoms namely dryness, grittiness or scratchiness, soreness or irritation, burning or watering, eye fatigue at the time of administration of the questionnaire, 72 hours and 3 months prior to administration of questionnaire. Each symptom was graded frequency (scale 0 to 3) and severity (scale 0 to 4) scores with 0 indicating infrequent or less severe symptom and higher scores indicating more frequent or severe symptoms. Composite scores were obtained by summing up these scores for a total of 28. The subject was categorized as normal, moderate or severe dry eye symptoms if the composite SPEED score was 0–6, 7–15, or 16–28, respectively. The scoring was done according to the standards indicated in literature [22]. In addition, participants' demographic profile, systemic and ocular history, trimester, gravidity (primagravida–first pregnancy; multigravida-pregnant more than once) were noted.

### 2.1. Data Analysis

Descriptive statistics were performed for demographic including age, systemic condition, trimester, and gravidity. Prevalence, frequency, and severity of the types of dry eye symptoms experienced were determined. Spearman correlation was used to study the relationship between speed score and age, trimester and gravidity. Binary logistic regression was used to identify the associated factors of dry eye symptoms such as age, gravidity, gestational age, systemic condition, and occupation in pregnant women. For 95% confidence level, *P* < 0.05 was considered statistically significant.

## 3. Results

Table 1 summarizes the distribution of the 428 participants in different age groups according to systemic condition, trimester, and gravidity. Most participants were of age group 21-30 years (77.8%), in their second or third trimester (78.3%), were of status primigravida (94.2%) and had normal systemic health (83.6%). Among the 428 participants, majority were house wives (*n* = 360), 12 were teachers, 13 were information technology personnel, and the remaining 43 were employed in other unorganized sector including tailors, assembly line workers etc.

### 3.1. Prevalence of Dry Eye Symptoms among Pregnant Women

**Figure 1:**
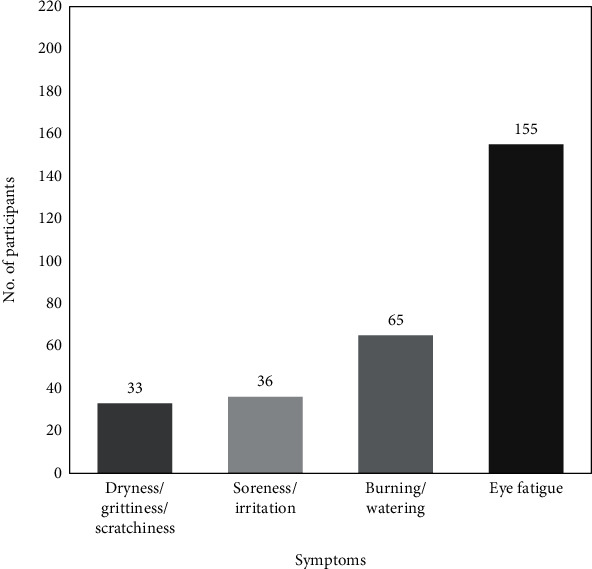
Distribution of dry eye-related symptoms in symptomatic participants (*n* = 203).

Among the 428 participants, at the time of administration of the questionnaire, 225 participants (52.6%) had no symptoms related to dry eye and 203 participants reported having one or more dry eye symptoms like dryness, grittiness or scratchiness, burning or watering, soreness or irritation, and/or eye fatigue. Among the 203 participants, only 50 participants (24.6%) reported having more than one symptom. There was no statistically significant difference (*P* = 0.41) in reported symptoms “at the visit” and for “within 72 hours”. Since, most participants were in second or third trimester, the data for “within past 3 months” symptoms are not included for analysis. Hence, the data at the time of administration is only presented here.  Figure 1 shows the distribution of symptoms among the participants; many have reported more than one symptom. At the time of administration of questionnaire, eye fatigue (*n* = 155,76.4%) was the most common symptom reported and dryness or itchiness or scratchiness was reported by 16.3% of the 203 participants.

### 3.2. Frequency of Dry Eye Symptoms Experienced by Pregnant Women

**Figure 2:**
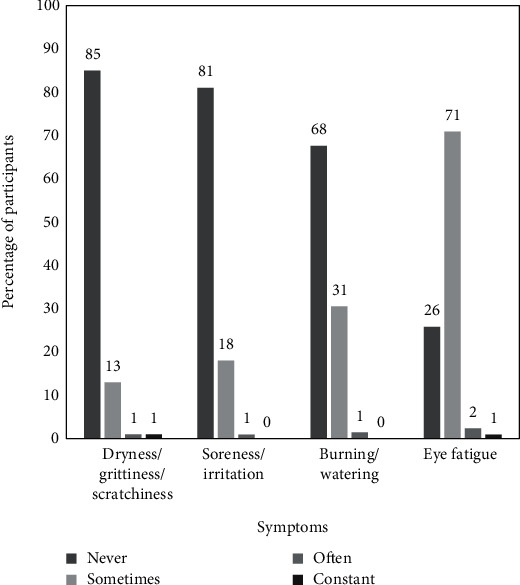
Frequency of occurrence of dry eye-related symptoms in symptomatic participants (*n* = 203).

Among those symptomatic (*n* = 203), the frequency of symptoms was classified in a four-point scale grading as “never”, “sometimes”, “often”, and “constant”.  Figure 2 shows the distribution of frequency of occurrence of the symptoms in participants. Less than 3% of the symptomatic participants had any of the symptoms present often or more. None of the symptoms were constantly present except for eye fatigue in 2 participants (1%).

### 3.3. Severity of Dry Eye Symptoms Experienced by Pregnant Women

**Figure 3:**
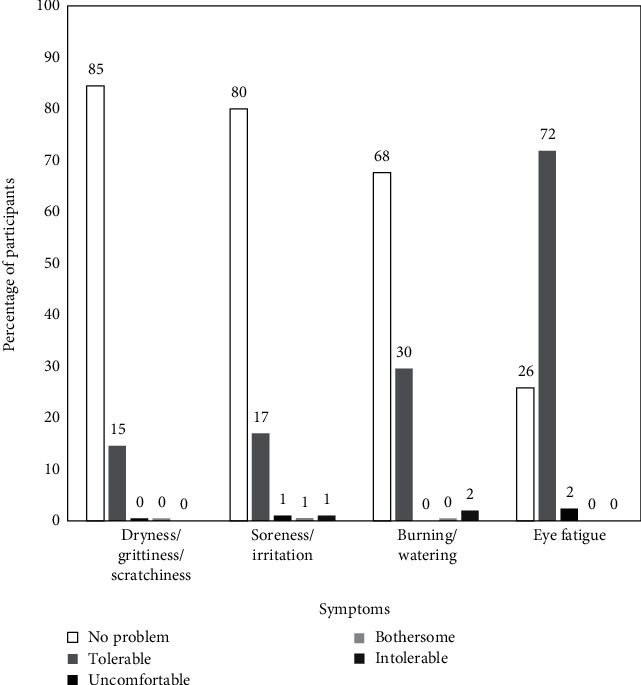
Severity of occurrence of dry eye-related symptoms in symptomatic participants (*n* = 203).

Among the symptomatic participants (*n* = 203), the severity of symptoms was classified in a five-point scale grading as “no problem”, “tolerable”, “uncomfortable”, “bothersome”, and “intolerable”. As seen from Figure 3, for most participants the presence of a symptom (other than eye fatigue) caused “no problem” (blank bars) or was “tolerable” (filled bars). Interestingly, presence of eye fatigue, was reported as “tolerable” by most participants (*n* = 151, 71.8%) while only 26% reported it to cause “no problem”. Except for burning or watering (*n* = 2, 1%) the presence of other symptoms was not intolerable.

Among the 225 asymptomatic participants, all were classified as “Normal” according to SPEED score cut off. Among the participants with dry eye-related symptoms only 5 (2.3%) of the participants had moderate dry eye score, and none were found to have severe dry eye score. No significant correlation was noted among SPEED score vs age (*r* = −0.02, *P* > .05), gravidity (*r* = −0.006; *P* > .05), or trimester (*r* = 0.38; *P* > .05). The symptom frequency score and severity score had a strong and significant correlation (*r* = 0.95, *P* < .001).

### 3.4. Factors Influencing Dry Eye Symptoms

Table 2 shows the results of univariate binary logistic regression. Factors such as age, gravidity, and gestational age did not significantly (*P* > 0.05) influence the occurrence of dry eye symptoms in pregnant women. Only coexisting systemic conditions (*P* = 0.047) such as diabetes mellitus, hypertension, thyroid disease, and occupational status (*P* = 0.01) of the participants were significantly associated with dry eye symptom occurrence. Concurrently, the Odd's Ratio was also significantly higher for women with preexisting systemic conditions (OR: 1.2) and employed women (OR: 1.67). To assess the effect of interaction between these predictors, a multivariate binary logistic regression was performed. Among the above indicated factors, only occupation was found to be significantly (*P* = 0.021, OR: 1.60; 95% CI: 1.07–2.28) influencing the dry eye symptoms.

## 4. Discussion

Recent studies show that dry eye disease is associated with hormonal changes [26, 27]. During pregnancy, the body undergoes several physiological hormonal changes which could potentially cause dryness [8]. Previous research shows that disruption of acinar cells, lacrimal dysfunction, fluctuations in blood glucose levels, and altered tear film physiology and associated ocular surface changes are some of the contributing factors towards dry eye in pregnant women [8–10, 28, 29]. While the health care delivery and monitoring systems are well established during pregnancy, the eye care and vision related symptoms are often neglected. In this study, we used the SPEED questionnaire to find the prevalence of subjective dry eye symptoms in pregnant women.

Specific questionnaires for evaluating dry eye-related symptoms and the quality of life in dry eye are available. In our study, we administered the SPEED questionnaire and found that around half of the pregnant women reported having dry eye-related symptoms, like dryness, grittiness or scratchiness, burning or watering, soreness or irritation, eye fatigue of varying frequency and severity, despite the decreased prevalence of dry eye diagnosis score (SPEED score). However, according to the SPEED score criteria, only 1.2% of pregnant women had subjective symptoms of dry eye (moderate dry eye score). A recent study, by Rizyal et al. reported that dry eye prevalence among pregnant women varies between 27.4% and 89.3% depending on the clinical criteria like tear breakup time and Schirmer's test used for dry eye diagnosis [30]. In fact, in a hospital-based cross-sectional study of pregnant women, 40.8% of dry eye disease was diagnosed with clinical dry eye tests and dry eye questionnaire [31]. While the presence of evaporative dry eye was not significantly associated with systemic parameters, they reported gestational age to be significantly associated [31]. The current study, on the other hand, observed that coexisting systemic conditions, such as diabetes, hypertension, and thyroid disease, were significantly associated with the occurrence of dry eye symptoms. Further to that, the participants' occupational status (*P* = 0.01) was found to be significantly related to the occurrence of dry eye symptoms. Further research is required to establish and comprehend the impact of occupational status on dry eye symptoms in pregnant women.

Ibraheem et al. reported a significantly lower Schirmer's readings in asymptomatic pregnant women compared to the controls [11]. While, the SPEED is a validated questionnaire for non-clinical evaluation of dry eye, the noncorrelational characteristic of the subjective symptoms and objective metrics should be considered in diagnosis and treatment of dry eye especially during pregnancy [14, 32].

Eye fatigue is a nonspecific symptom associated with several factors like improper sleep, uncorrected refractive error, or binocular vision anomalies [33–35]. Similar to the observations made by Toda et al. eye fatigue was the most widespread symptom reported by pregnant women [34]. Interestingly, sleep disturbances and fluctuations in refraction are not uncommon during pregnancy [29, 36]. Furthermore, during pregnancy clinical features of dry eye can be exacerbated with preexisting ocular condition such as associated contact lens wear. The SPEED questionnaire did not include these aspects in dry eye symptom evaluation. It would be of great interest and importance to do a comprehensive evaluation including subjective and objective tests to diagnose the actual prevalence of dry eye in pregnant women and derive guidelines for eye care during pregnancy.

In our study, the participants in their third trimester reported more symptoms, however not with statistical significance. In an earlier study by Nkiru et al., using OSDI questionnaire and using a cut off score of 13, around 78.5% had dry eye [37]. This increase in prevalence in the third trimester was consistent with diagnosis using objective clinical evaluation [28]. Furthermore, Skare et al. study concluded that pregnant women suffer more from tear dysfunction than nonpregnant women with prevalence of tear dysfunction stronger in higher parity [10]. In this study, more participants in their third trimester reported symptoms (*n* = 84) than those in other trimester. However, a statistically significant association between occurrence of dry eye symptoms and trimester was not found. A modified diagnosis criterion could show results that are both clinically and statistically relevant.

The SPEED questionnaire used in this study was tested for the conceptual and semantic equivalence with Indian population; none of the participants in our study faced any difficulty while responding to the questionnaire. Administration of the SPEED questionnaire consumed less than five minutes of time. Strikingly, none of symptomatic participants (48.5%) were under any type of treatment to alleviate their symptoms, possibly due to the nonsevere nature of the symptoms. In a study by Doughty et al., subjective prevalence of dry eye was assessed in 30 successive volunteers presenting to optometric practices for various reasons and they reported 29% prevalence in dry eye symptoms [38]. These results, nevertheless, emphasize the gap in symptom and eye care provided for pregnant women. Identifying and treating dry eye has been shown to improve quality of life [39, 40]. Educating pregnant women on the impact of physiological imbalance on dry eyes and providing eye care recommendations/referrals along with general health monitoring, will further improve quality of life in pregnant women.

## 5. Conclusion

The prevalence of dry eye-related symptoms was found in about half of the surveyed pregnant women. Due to decreased severity of the symptoms and the infrequent occurrence of these symptoms, SPEED score-based dry eye diagnosis was found to be less among pregnant women. No significant difference was seen in symptoms or SPEED score among type of pregnancy, trimester, and systemic condition.

## Figures and Tables

**Table 1:** Number of participants in each age group, percentage of participants with systemic conditions (DM: Diabetes Mellitus, HTN: Hypertension, and TD: Thyroid Disease), trimester and gravidity.

Age group (years)	Systemic condition (%)					Trimester (%)			Gravidity (%)	
	Normal	Only			DM/TD/HTN	I	II	III	Primigravida	Multigravida
		DM	TD	HTN						
<20 (*n* = 26)	84.6	—	15.4	—	—	26.9	50.0	23.1	100.0	—
21-25 (*n* = 149)	89.9	2.7	6.7	—	0.7	20.1	40.9	38.9	98.7	1.3
26-30 (*n* = 184)	85.3	6.0	6.5	1.1	1.1	21.7	39.1	39.1	91.8	8.2
31-35 (*n* = 52)	63.6	3.8	26.9	3.8	1.9	23.1	32.7	44.2	96.2	3.8
36-40 (*n* = 14)	64.3	21.4	—	—	14.3	21.4	50.0	28.6	64.3	35.7
41-45 (*n* = 3)	100	—	—	—	—	33.3	33.3	33.3	66.7	33.3

**Table 2:** Univariate binary logistic regression.

Parameter	*χ* ^2^	*P* value	Odds ratio	95% confidence interval
Age	0.3489	0.555	0.9872	0.9457 - 1.0305
Gravidity	0.0425	0.8367	0.9185	0.4092 - 2.0617
Gestational age	0.9181	0.3384	1.1303	0.8796 - 1.4525
Systemic condition	3.7765	0.0472	1.2009	0.9871 - 1.4611
Occupation	6.6444	0.0103	1.6698	1.1287 - 2.4704

## Data Availability

The database generated and/or analysed during the current study are not publicly available due to privacy, but are available from the corresponding author on reasonable request.
